# Breathing-Controlled Electrical Stimulation (BreEStim) Selectively Modulates Affective and Cognitive Components of Pain—An EEG Study

**DOI:** 10.3390/bioengineering13050501

**Published:** 2026-04-25

**Authors:** Ahmad Z. Rao, Michael Houston, Hao Meng, Shengai Li, Yingchun Zhang, Sheng Li

**Affiliations:** 1 Department of Physical Medicine and Rehabilitation, University of Texas Health Science Center at Houston, Houston, TX 77030, USA; ahmad.z.rao@uth.tmc.edu (A.Z.R.); hao.meng@uth.tmc.edu (H.M.); shengai.li@uth.tmc.edu (S.L.); 2 TIRR Memorial Hermann, Houston, TX 77030, USA; 3 Department of Biomedical Engineering, University of Miami, Coral Gables, FL 33146, USA; mxh1983@miami.edu (M.H.); y.zhang@miami.edu (Y.Z.); 4 Desai Sethi Urology Institute, University of Miami, Miami, FL 33136, USA; 5 Miami Project to Cure Paralysis, University of Miami, Miami, FL 33136, USA

**Keywords:** breathing-controlled stimulation, cortical processing, event-related potentials, pain modulation

## Abstract

Breathing-controlled electrical stimulation (BreEStim) is an innovative neuromodulation intervention that synchronizes deep voluntary breathing with peripheral electrical stimulation. Prior studies have shown its analgesic effects in healthy adults and spinal cord injury patients with neuropathic pain. The present study used EEG to examine BreEStim’s neural effects on sensory, affective, and cognitive components of pain. Fourteen healthy participants (7 M, 7 F) completed 30 min of BreEStim and conventional electrical stimulation (EStim) interventions in a randomized, crossover within-subject design. Electrical pain thresholds (EPT) and EEG were recorded pre- and post-intervention. Event-related potentials (ERPs) at pre-EPT-level stimuli before and immediately after each intervention were analyzed for early sensory (P30) and affective (P250) processing, while resting-state EEG assessed spectral power across delta, theta, alpha, and beta bands for cognitive processing. Both BreEStim and EStim increased EPT, indicating short-term habituation. There was no change in early ERP responses (P30) after each intervention, suggesting preserved sensory perception. BreEStim selectively reduced P250, reflective of the affective component of pain. BreEStim significantly increased delta and theta band power and reduced alpha band power on resting-state EEG analyses, whereas no significant changes after EStim were observed. Collectively, BreEStim preserves sensory encoding while selectively modulating affective and cognitive dimensions of pain, supporting its potential as a targeted, non-pharmacological neuromodulation strategy.

## 1. Introduction

Pain is a multidimensional experience encompassing sensory-discriminative, affective-motivational, and cognitive-evaluative components that are anatomically and phenomenologically separable yet interact in complex ways [1,2]. While specific interventions can theoretically target individual dimensions—analgesics for sensory aspects, psychological therapies for affective suffering, and cognitive-behavioral strategies for evaluative processes—complete, selective management of each dimension in isolation is rarely achieved in clinical practice [3]. Most interventions affect multiple dimensions simultaneously, and current clinical tools do not robustly distinguish or manage each dimension independently. Chronic and neuropathic pain conditions affect approximately 25% of Americans and often prove resistant to conventional pharmacological interventions [4]. The limitations of current strategies, including concerns about opioid dependence and incomplete efficacy, have intensified the search for novel non-pharmacological approaches [5]. Best practice guidelines now advocate for integrated, multimodal strategies that acknowledge the interplay between all pain dimensions [6], highlighting the need for innovative interventions that can address multiple aspects of pain simultaneously.

Neuromodulation techniques have emerged as promising alternatives for pain management, leveraging the nervous system’s inherent plasticity to alter pain perception and processing. The neurophysiological effects of neuromodulation techniques can be captured in EEG patterns, which may serve as both a mechanistic probe and a potential biomarker for optimizing individualized pain treatment strategies. Transcutaneous electrical nerve stimulation (TENS) has been shown to modulate pain-evoked potentials, with effective stimulation paradigms reducing N2-P2 amplitudes and altering the distribution of cortical activation away from pain-processing regions [7,8]. However, its effects are often variable, and the underlying mechanisms remain incompletely understood [9,10,11]. Studies of repetitive transcranial magnetic stimulation (rTMS) targeting the motor cortex have demonstrated normalization of aberrant alpha and theta power in chronic pain patients, with these changes correlating with clinical pain relief [12,13,14]. Transcranial direct current stimulation (tDCS) modulates oscillatory activity in pain-relevant frequencies, particularly increasing alpha power and reducing theta activity in prefrontal and sensorimotor regions, effects that persist beyond the stimulation period and parallel improvements in pain ratings [15,16]. Importantly, responders to neuromodulation treatments often show distinct baseline EEG characteristics compared to non-responders, suggesting that pre-treatment neural signatures may help predict therapeutic outcomes [17]. These findings collectively indicate that EEG can capture the neurophysiological effects of neuromodulation and may serve as both a mechanistic probe and a potential biomarker for optimizing individualized pain treatment strategies.

Breathing-controlled electrical stimulation (BreEStim) represents an innovative neuromodulation approach that capitalizes on brain–body interaction by synchronizing peripheral electrical stimulation with voluntary breathing patterns [18]. Unlike conventional stimulation paradigms, BreEStim specifically targets the temporal relationship between respiration and neural processing, engaging cortical and subcortical networks involved in pain modulation. Our previous work has demonstrated the analgesic efficacy of BreEStim in healthy adults [19,20] and in patients with spinal cord injury experiencing neuropathic pain [21,22]. Critically, these effects are not attributable to breathing exercises alone [23] but emerge specifically from the temporal coupling of voluntary respiration and peripheral stimulation, highlighting a unique mechanistic pathway. Furthermore, BreEStim has been shown to restore autonomic function in spinal cord injury patients [24], suggesting that its therapeutic effects involve integrated modulation of both central pain-processing networks and autonomic regulatory systems.

Despite these established behavioral and autonomic outcomes, the cortical mechanisms through which BreEStim modulates pain perception remain largely unexplored. EEG offers a means to characterize these mechanisms by capturing both rapid event-related potentials (ERPs) and baseline oscillatory states. Early ERP components such as P30 reflect sensorimotor integration, primarily in the cortical somatosensory representation of the stimulus surface, whereas later components such as P250 reflect the affective appraisal of a nociceptive stimulus [25]. The bilateral activity from cortical sources such as the anterior cingulate cortex and insula for the affective component of pain responses is typically maximal at the vertex, located at the Cz position [26]. Resting-state oscillations across EEG frequency bands further reflect hierarchical mechanisms of pain processing, with delta–theta activity indexing thalamocortical dysrhythmia and affective–motivational processing [27], alpha reflecting impaired cortical inhibition [28], beta indicating altered sensorimotor integration [29], and gamma encoding local cortical synchrony and subjective pain intensity [30].

Accordingly, this study compared pain-related cortical activities between conventional electrical stimulation (EStim) and BreEStim by assessing changes in P30 and P250 responses to nociceptive stimuli, along with resting-state spectral dynamics. We hypothesized that, as compared to EStim, BreEStim would (1) not differ in early sensorimotor processing reflected by similar P30 amplitude, (2) reduce affective–attentional engagement reflected by diminished P250 amplitude, and (3) shift resting-state oscillations toward patterns indicative of reduced cortical hyperexcitability.

## 2. Materials and Methods

### 2.1. Subjects

Fourteen (14) individuals (7 males, 7 females; age: 31.8 ± 9.9 years) participated in this study. The participants were healthy and did not have a history of neuromusculoskeletal impairments or injury. No female participants were pregnant. The experiments conformed to the Declaration of Helsinki and were approved by the Institutional Review Board (IRB) at the University of Texas Health Science Center at Houston. Informed consent was obtained from all participants.

### 2.2. Study Design

**Figure 1:**
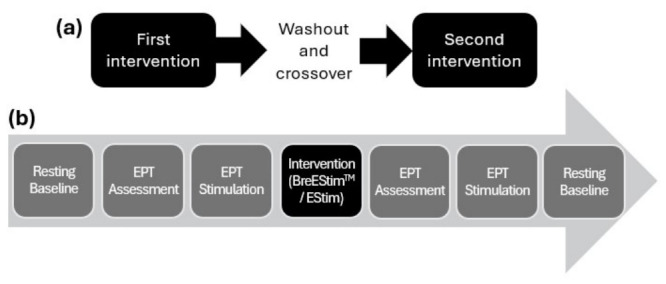
The experimental procedure showing (**a**) the overall design and (**b**) the task sequence on each intervention day. EPT: electrical pain threshold, BreEStim^TM^: breathing-controlled electrical stimulation, EStim: electrical stimulation.

This study was a randomized crossover trial with two interventions, namely EStim and BreEStim^TM^. The order of the interventions was randomly assigned, ensuring that by the end of the study, half of the participants started with either intervention. For each participant, both interventions took place with at least a three-day washout period, as shown in Figure 1a.

### 2.3. Procedure

Figure 1b illustrates the experimental procedure in the sequence of tasks that were conducted on each testing day. The participants were seated comfortably in a well-ventilated and quiet room with their arms supported and hands supinated on a desk. Their resting EEG (rsEEG) was recorded for 5 min in an eyes-closed state at the beginning of the experiment. The electrical pain threshold (EPT) assessments and EEG responses to EPT stimulation were measured immediately before and after the intervention. The EPT assessments were carried out separately on each testing day. Lastly, the rsEEG was measured again ~10 min post-intervention.

**Figure 2:**
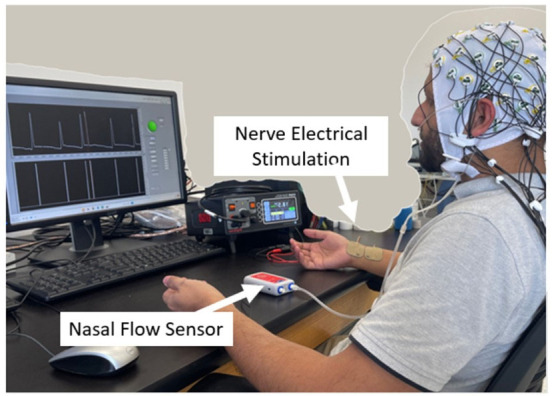
The experimental setup showing the electrical stimulator, wrist electrodes, nasal cannula, flow sensor, EEG cap, and control software.

The participants were prepared for an electroencephalogram (EEG) recording with 64 electrodes mounted, according to the 10–10 international system, on a cap. The reference electrode was positioned at the Cz site, and the ground electrode was placed at Fpz. The electrode impedance was kept below 5 kΩ, and the sampling rate of the EEG machine (actiCHamp Plus, Brain Vision LLC, Morrisville, NC, USA) was kept at 2500 Hz. The experimental setup is shown in Figure 2.

For EPT assessments, a pair of trimmed surface electrodes ~1 cm × 1 cm, separated by ~1 cm, were placed bilaterally over the median nerve, ~1 cm above the wrist. Participants’ electrical pain thresholds were determined separately for each side by gradually increasing the current intensity delivered by the constant current stimulator (DS8R, Digitimer Ltd., Hertfordshire, UK) in increments of ~1 mA until they reported a pain level of 1 according to the Visual Analog Scale (VAS). Three trials of EPT assessment were performed, and the average EPT value was calculated. The average EPT value from the right median nerve was used to provide 20 electrical current stimulations, randomly at 4–6 s apart, on the right median nerve, before and after the interventions. It is important to note that the same intensity of electrical current (i.e., pre-intervention EPT) was used to measure event-related potentials before and after the intervention (Figure 1b). Time-locked markers for EPT stimulations were sent to the EEG recording software (BrainVision Recorder, version 1.27.0001). The average EPT value from the left median nerve was used as the initial stimulation level during the interventions.

### 2.4. Interventions

To set up the interventions, the electrode placed for the EPT assessment over the left median nerve was used to deliver a 0.1 ms square wave stimulation at EPT current intensity. This intensity of the stimulation was manually gradually increased to a painful yet tolerable pain level (6~7 on the VAS) by the participants. During both sessions, participants received multiple single electrical stimuli consisting of 0.1 millisecond square waves through a customized LabVIEW (version 2024, National Instruments, Austin, TX, USA) software program. However, to maintain consistent pain perception during the sessions, participants were reminded every 50–60 stimulations to increase the electrical stimulation intensity if they felt a reduction in pain level. The painful electrical stimulation intensity and session duration, which was ~30 min, were similar across both interventions for each participant. For both interventions, participants were regularly asked about any incidence of fatigue and/or adverse effects that required rest or cessation of the experiment.

The interventions differed in only their control mechanisms. During EStim, the stimuli were delivered at random intervals ranging from 3 to 5 s, with the total number of stimuli matched between interventions or maintained at approximately 400 counts when EStim was performed as the first condition. During BreEStim, additionally, a nasal cannula was worn by the participants that connected to an airflow meter (Ultima Pressure Sensor Kit 0580, Braebon Medical Corporation, Kanata, ON, Canada). The participants were initially asked to take a few deep breaths to record their maximal voluntary inhalation airflow. They were then instructed to breathe using fast, strong, and deep inhalations. As the airflow during these breaths reached the pre-set threshold of 40% of their maximal voluntary inhalation airflow value, electrical stimulation was triggered via the current stimulator. The breathing pattern was visually monitored, and verbal feedback was provided to participants to ensure consistency and match the number of stimuli across both interventions.

### 2.5. Data Processing

All EEG data were preprocessed using the EEGLAB toolbox in MATLAB (version R2024b, MathWorks, Natick, MA, USA) [31]. Signals were downsampled to 250 Hz and filtered using basic finite impulse response filters, with a 1–30 Hz band-pass to attenuate low-frequency drifts and high-frequency noise, followed by a 60 Hz notch filter to remove powerline interference. Data were re-referenced to the common average reference, calculated by subtracting the mean signal across all electrodes. EEG traces were visually inspected across all channels for non-physiological artifacts, including bursts of irregular high-amplitude deflections, sudden spikes, and flatlines. Trials and EEG segments contaminated by artifacts, representing no more than 10% of the data, were manually excluded from analysis. Channels exhibiting abnormal activity, such as poor connections, excessive noise, flat signals due to disconnections, or high impedance, were marked as bad (no more than two channels per participant) and interpolated using spherical spline interpolation. Finally, independent component analysis (ICA) was applied to remove residual ocular, cardiac, and muscle artifacts.

For rsEEG, the time domain data were first normalized by applying a z-score transformation to each channel independently. This procedure sets each channel to have a zero mean value and unit variance, facilitating comparison across participants and recording sessions. The power spectral density (PSD) of the normalized data was calculated using Welch’s method in MATLAB. A sliding Hanning window of 512 samples with 50% overlap was used to enhance estimation quality by controlling spectral leakage and data variance. The power was computed within each frequency band (delta: 1–4 Hz, theta: 4–8 Hz, alpha: 8–12 Hz, and beta: 13–30 Hz) for all EEG channels. To observe the ongoing cognitive modulation of pain, the dorsolateral prefrontal cortex (DLPFC) activity was monitored over F3 and F4 channel locations [32]. The PSD from the averaged activity from these channels was calculated.

The epochs for event-related potential (ERP) analysis were extracted from −100 ms to +1000 ms centered on the EPT stimulation marker. Twenty stimulation trials per participant were included in the analysis. As recordings were acquired under a controlled experimental protocol with minimal movement and time-locked peripheral stimulation, no explicit trial rejection was performed, and all trials were retained following preprocessing. Baseline correction was performed by averaging the signal during the 100 ms pre-stimulus interval and subtracting this mean from the entire epoch to correct for voltage offsets and slow drifts.

The reliability of the P30 and P250 components was ensured by averaging 20 time-locked stimulation trials per participant, which suppresses non-phase-locked EEG noise and enhances stimulus-locked responses. Peak amplitudes were then quantified within predefined latency windows and electrode sites based on prior somatosensory ERP literature, thereby minimizing subjective peak selection. Subsequently, grand averaging across all participants was performed to obtain the group-level ERP waveform. Although a formal signal-to-noise ratio was not computed, this ensemble averaging approach reduces non-time-locked background EEG activity and improves the detectability of stimulus-locked components.

CP3, located contralateral to the right wrist stimulation, was included to capture potential contributions from the somatotopically organized contralateral somatosensory region (S1) [33]. At CP3, the ERP peak that falls within the 20~40 ms window post-stimulus (P30) was analyzed for initial sensory perception. The ERP peak within the 200~300 ms window post-stimulus (P250) was quantified for somatosensory processing at Cz.

### 2.6. Statistical Analysis

Descriptive statistics were used for demographics. Separate two-way repeated measures ANOVAs were used with the independent variables being intervention (factors: BreEStim, EStim) and time (factors: pre, post). The dependent variable was the peak amplitude when analyzing the ERPs (at P30 and P250), while it was the band-average PSD value when analyzing the resting EEG across frequency bands. A three-way repeated measures ANOVA was used to compare the pain thresholds with the factors intervention, time, and hand (factors: right, left). Post hoc analyses were performed to control the family-wise error rate using the Bonferroni-corrected pairwise comparisons for significant main effects. For significant interaction effects, follow-up paired-samples t-tests were conducted to determine the direction of the interaction. All statistical tests were performed in SPSS, and the level of significance was kept at 0.05 for all tests.

## 3. Results

### 3.1. Electrical Pain Thresholds

All participants tolerated the stimulation procedures well and completed both BreEStim and EStim sessions without adverse effects. The bilateral electrical pain thresholds (EPT) were determined separately prior to each intervention. The left side was then used for the intervention, whereas the right side was used to deliver EPT-level electrical pain stimulations. During the stimulation sessions, the electrical current intensity was individually adjusted according to each participant’s EPT level. Each session lasted approximately 30 min and consisted of (mean ± standard deviation) 417.2 ± 90.4 stimuli with an average EStim/BreEStim stimuli ratio of 1.004 ± 0.07.

**Figure 3:**
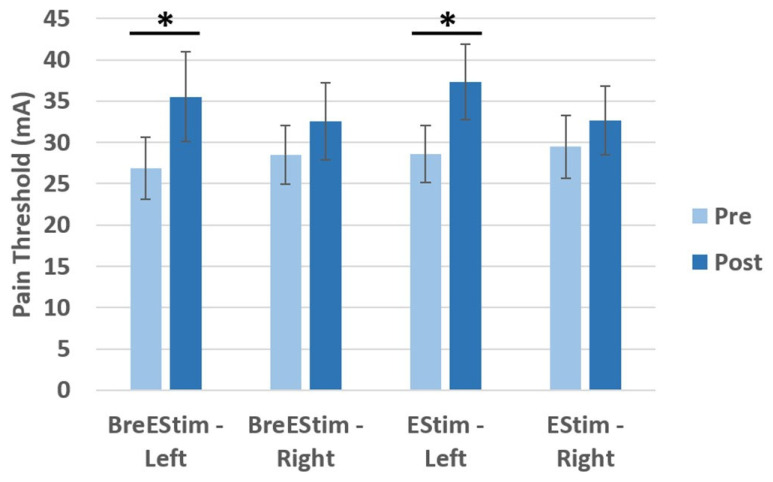
Electrical pain threshold levels before (pre) and after (post) BreEStim and EStim interventions. * *p* < 0.001.

Table 1 presents the results of the three-way ANOVA, which revealed a significant main effect of Time (F(1,13) = 11.38, *p* = 0.005, ηp^2^ = 0.467) representing a medium-to-large effect and a large and significant Time × Hand interaction (F(1,13) = 55.54, *p* < 0.001, ηp^2^ = 0.810), indicating that changes in EPT over time differed substantially between hands. The EPT, as shown in Figure 3, increased more in the left hand (mean ± standard error; BreEStim: 26.89 ± 3.57 mA to 35.54 ± 5.40 mA; EStim: 28.62 ± 3.45 mA to 37.33 ± 4.57 mA) than in the right hand (BreEStim: 28.46 mA ± 3.34 to 32.55 mA ± 4.67; EStim: 29.46 ± 3.79 mA to 32.66 ± 4.20 mA). Bonferroni-corrected pairwise comparisons for Time x Hand interaction showed no significant change in EPT for the right hand between pre- and post-intervention (mean difference MD = –3.65, SE = 1.83, *p* = 0.068). However, the left hand demonstrated a significant increase in EPT from pre- to post-intervention (MD = 8.69, SE = 1.89, *p* < 0.001), indicating a selective effect of the intervention on the stimulated hand.

### 3.2. Event-Related Potentials and Cortical Responses

ERP analysis was performed to examine the temporal dynamics of cortical responses to peripheral painful electrical stimulation and to compare the changes induced by BreEStim and EStim interventions. This analysis focused on two electrode sites: CP3, corresponding to contralateral primary somatosensory cortex activity (early sensory processing), and Cz, reflecting midline cortical structures involved in higher-order pain modulation and attentional processing. Table 2 shows the results obtained from four separate two-way repeated measures ANOVAs for different components of the ERP waveforms. The time effect was significant in all peaks except P30, whereas the interaction effect was only significant for P250 at Cz.

Pairwise comparisons using Bonferroni adjustment for the significant Intervention x Time interaction indicated that P250 ERP at Cz significantly decreased from Pre to Post in BreEStim (MD = 2.20, SE = 0.69, *p* = 0.008; mean ± standard error; pre: 9.93 ± 1.65 µV; post: 5.74 ± 0.81 µV), whereas no significant change was observed in EStim (MD = 0.17, SE = 0.49, *p* = 0.733; pre: 9.84 ± 1.20 µV; post: 8.45 ± 1.23 µV). There was no significant change in P30 ERPs at CP3 after both interventions, suggesting that cortical sensation of peripheral stimulation was not changed after BreEStim or EStim interventions.

**Figure 4:**
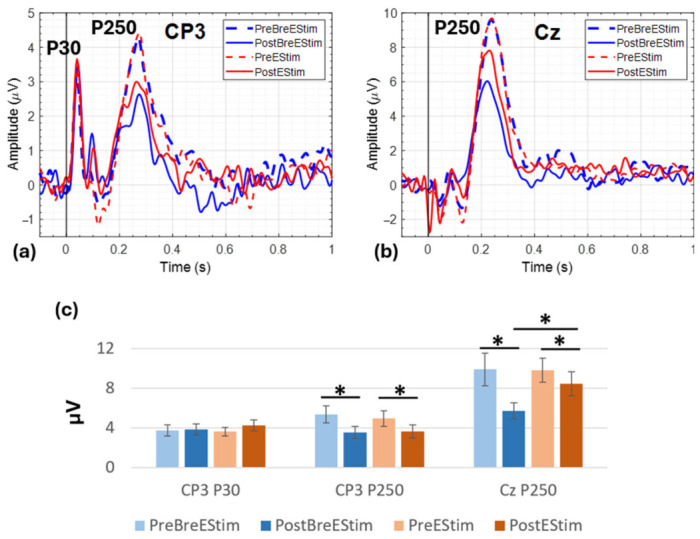
The averaged ERPs at (**a**) CP3 and (**b**) Cz, and (**c**) the bar graphs representing mean and standard error of the peak amplitudes. The significance is indicated as * *p* < 0.05.

The averaged ERP waveforms during electrical stimulations for each of the four conditions—Pre-BreEStim (PreB), Post-BreEStim (PostB), Pre-EStim (PreE), and Post-EStim (PostE)—are shown in Figure 4. The ERP at CP3, contralateral to the stimulations on the right wrist, is shown in Figure 4a. The ERP at the central electrode Cz is shown in Figure 4b. The peaks P30 and P250 are marked on the plots. The pre-intervention ERP waveforms were found to be similar in peak amplitude and topology for both interventions at CP3 and Cz, whereas the post-intervention waveforms were similar for P30 at CP3 but reduced for P250 at both CP3 and Cz. To further visualize the direction and magnitude of modulation, ERP peak amplitudes were also expressed as bar graphs, as shown in Figure 4c, with the error bars representing the standard error. The peak amplitudes for P30 at CP3 were BreEStim (mean ± standard error; pre: 3.75 ± 0.57 µV; post: 3.84 ± 0.55 µV) and EStim (pre: 3.62 ± 0.42 µV; post: 4.25 ± 0.53 µV), whereas for P250 at CP3 they were BreEStim (mean ± standard error; pre: 5.35 ± 0.86 µV; post: 3.54 ± 0.59 µV) and EStim (pre: 4.94 ± 0.80 µV; post: 3.65 ± 0.65 µV). Together, these findings indicate that BreEStim significantly reduced the P250 at Cz as compared to EStim.

### 3.3. Resting-State EEG

**Figure 5:**
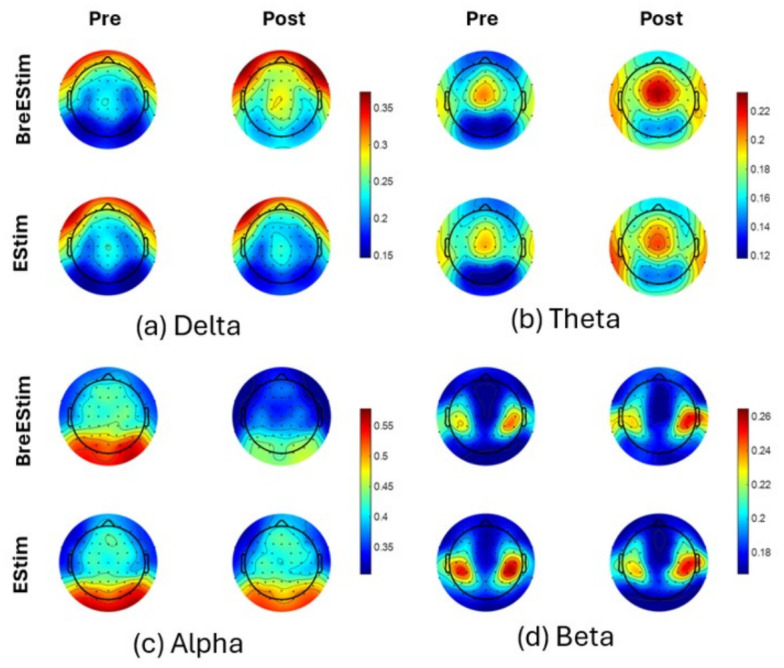
Topoplots representing (**a**) delta, (**b**) theta, (**c**) alpha, and (**d**) beta band power of all channels in pre (first column) and post (second column) for BreEStim (first row) and EStim (second row). The color bar axes are constant within each frequency band and represent arbitrary units.

The topoplots of averaged power across participants and trials for the five different frequency bands from the resting-state EEG data for all channels are shown in Figure 5. For each frequency band subplot, the first row represents BreEStim, while the second represents EStim; the first column shows pre-intervention activity, while the second column shows post-intervention activity. There is a symmetrical trend of activity across the scalp in all frequency bands. The delta (Figure 5a) and theta (Figure 5b) bands show a global increase, whereas the alpha (Figure 5c) band shows a global decrease in activity post-BreEStim. The beta band (Figure 5d) shows similar activity before and after both interventions. For EStim, all the frequency bands show similar activity pre- and post-intervention, except for theta (Figure 5b), where the global activity increased post-intervention.

**Figure 6:**
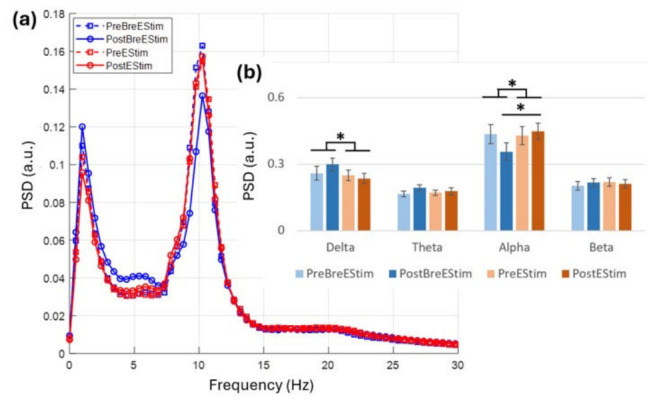
(**a**) Power spectrum of resting-state EEG showing mean power over channels F3, F4 for Pre-/Post-BreEStim/EStim. (**b**) The bar graphs represent the average power in each condition with standard error bars and significance indicated as * *p* < 0.05.

The DLPFC activity was computed by averaging the signals from the F3 and F4 channels. Figure 6a presents the power spectral density (PSD) across the frequency range for the pre- and post-intervention conditions. Table 3 summarizes the results of the separate two-way repeated-measures ANOVAs conducted for each frequency band. Significant effects were observed in the delta, theta, and alpha bands.

The delta-power ANOVA revealed a significant main effect of Intervention, F(1,13) = 5.702, *p* = 0.033, ηp^2^ = 0.305, with higher delta PSD during BreEStim than EStim overall. Neither the main effect of Time nor the Intervention × Time interaction was significant. The theta-power ANOVA had a significant main effect of Time, F(1,13) = 5.806, *p* = 0.032, ηp^2^ = 0.309; however, the main effect of Intervention or the Intervention × Time interaction was not significant.

The alpha-power ANOVA showed a significant main effect of Intervention (F(1,13) = 5.987, *p* = 0.032, ηp^2^ = 0.352) and a significant Intervention × Time interaction (F(1,13) = 8.104, *p* = 0.016, ηp^2^ = 0.424), whereas the main effect of Time was not significant (F(1,13) = 0.934, *p* = 0.355). Post hoc comparisons indicated that alpha activity was similar between BreEStim and EStim at Pre (*p* = 0.480; BreEStim = 0.435 ± 0.043 a.u., EStim = 0.428 ± 0.041 a.u.), but at Post, alpha was lower in the BreEStim condition (*p* = 0.019; BreEStim = 0.357 ± 0.040 a.u., EStim = 0.449 ± 0.036 a.u.). Within-condition changes from Pre to Post were not significant for either BreEStim (*p* = 0.119) or EStim (*p* = 0.216). Figure 6b represents these changes in average power using bar graphs. These findings suggest that the intervention effect on alpha emerged only after the intervention, driven by a post-intervention decrease in alpha activity in the BreEStim condition.

## 4. Discussion

The present study investigated the cortical mechanisms underlying pain processing between BreEStim and EStim, using EEG to characterize changes in both evoked responses to nociceptive stimuli and resting-state cortical dynamics. Building on prior work demonstrating the behavioral and autonomic efficacy of BreEStim in healthy adults and spinal cord injury patients [19,20,21,24], the novelty of this study lies in its effort to elucidate the neurophysiological substrates underlying these therapeutic effects and the mechanisms underlying the analgesic effects of BreEStim.

### 4.1. Short-Term Pain Habituation

Both BreEStim and EStim produced an immediate increase in pain thresholds on the stimulated hand, consistent with short-term pain habituation. Because the post-intervention pain threshold was assessed immediately after stimulation (less than 3 min), the behavioral change likely reflects rapid habituation mechanisms rather than sustained analgesia. Behavioral habituation following EStim likely reflects subcortical mechanisms, including spinal gating and brainstem-mediated descending inhibition, with additional contributions from attentional engagement or expectancy effects [34]. Supporting this, resting-state EEG (rsEEG) showed a global increase in theta power for both interventions, suggesting modest engagement of top-down cortical networks even with EStim [35]. Importantly, the rsEEG was recorded after a ~10 min interval following the intervention, which is similar timing to that of the assessment in the previous studies where analgesic effects of BreEStim were reported in healthy and spinal cord injury subjects [19,20,21,24]. Thus, the rsEEG results are more likely to reflect stabilized neural states associated with the analgesic process rather than the immediate habituation observed behaviorally.

In contrast, BreEStim uniquely produced increases in delta power and decreases in alpha power, indicating more extensive cortical network reorganization that may facilitate modulation and enhance processing of nociceptive input. Together, these findings suggest that while both interventions can induce short-term habituation at the behavioral level, BreEStim additionally engages cortical circuits underlying early sensory integration and later affective–cognitive evaluation, which may contribute to the development of more sustained analgesic effects.

### 4.2. Differential Modulation of Pain-Processing Stages

Consistent with our hypotheses, BreEStim and EStim elicited similar early sensory responses, as reflected in comparable P30 amplitudes. The P30 component, generated in frontal motor areas and reflecting sensorimotor integration [25], appears to be driven primarily by the electrical stimulation parameters themselves rather than by the temporal relationship with respiration. This preservation of early somatosensory processing indicates that both interventions engage initial nociceptive pathways to a similar degree, suggesting that the peripheral afferent input and primary sensory encoding are not fundamentally altered by BreEStim.

In marked contrast, BreEStim evoked a significant reduction in P250 amplitude compared to EStim. The P250 component has been associated with affective evaluation and attention allocation to painful stimuli, with neural generators likely involving the anterior cingulate cortex and secondary somatosensory areas [36]. The reduction here suggests that BreEStim specifically modulates higher-order appraisal and affective-motivational processing of nociceptive input. The selective attenuation of this later component while preserving early sensory responses indicates that BreEStim’s principal mechanism operates not at the level of peripheral gating or initial afferent processing, but rather during the later stage where pain acquires its emotional salience and motivational significance.

### 4.3. Alterations in Resting-State Cortical Dynamics

Beyond event-related responses, rsEEG was recorded in an eyes-closed condition several minutes after the intervention. The results indicated that compared to EStim, BreEStim induced a distinctly different resting state, characterized by increased activity in delta and theta power and reduced alpha activity. These spectral shifts suggest an altered cortical network configuration induced by BreEStim.

The presence of measurable changes in rsEEG indicates that BreEStim induces relatively sustained alterations in cortical dynamics. Because the recordings were obtained with a delay after the intervention, these findings likely reflect aftereffects on cortical networks rather than the instantaneous state during BreEStim itself.

The increase in slow-wave activity may appear counterintuitive, as chronic pain states are often associated with elevated theta oscillations linked to thalamocortical dysrhythmia [37]. However, the functional significance of theta activity depends strongly on its context and spatial distribution, as elevated theta in chronic pain is usually observed in the frontal region [38,39]. By contrast, in healthy individuals exposed to experimentally induced pain [38], similar theta increases may reflect adaptive, transient network responses, supporting top-down modulation and coordination of large-scale cortical networks.

In the present study, theta power increased following both BreEStim and conventional EStim, suggesting engagement of shared regulatory mechanisms rather than a pathological dysrhythmic state. Such theta increases have been associated with enhanced top-down modulation and internally focused processing states, including those observed during attention regulation and meditative practices [40]. Moreover, slow-wave activity during wakefulness has been linked to reduced cortical excitability and diminished responsiveness to external stimuli, which may facilitate short-term pain habituation by reducing the salience of nociceptive input.

The concurrent reduction in alpha power observed specifically following BreEStim is particularly informative given alpha’s established role in cortical inhibition and sensory gating. While increased alpha activity in sensorimotor regions is often associated with reduced pain processing [41], alpha suppression can also reflect active engagement of attentional and regulatory networks [42,43]. In the present study, this alpha suppression was also prominent over the DLPFC, a key hub for top-down cognitive control [44], likely reflecting sustained recruitment of executive networks that regulate attentional focus and the affective appraisal of pain and suggesting that BreEStim specifically engages cortical networks involved in controlling the subjective experience of pain.

Collectively, the combination of increased slow-wave activity and decreased alpha power suggests that BreEStim induces a distinctive cortical state that differs from both chronic pain–related dysrhythmia and the oscillatory patterns typically associated with passive analgesia. Rather than simply reversing pathological oscillations or enhancing inhibitory processes, BreEStim may promote a novel network configuration characterized by enhanced top-down control and reduced stimulus salience, thereby supporting sustained modulation of pain processing.

### 4.4. Clinical Implications and Therapeutic Potential

The neurophysiological profile observed in this study has important implications for the therapeutic application of BreEStim. The selective modulation of cognitive–affective pain processing, alongside preservation of early sensory encoding, suggests that BreEStim may be particularly effective for conditions in which affective distress and attentional bias toward pain contribute substantially to disability, such as neuropathic and nociplastic pain syndromes [45]. By reducing emotional salience and cognitive elaboration without abolishing sensory awareness, BreEStim may alleviate suffering while maintaining protective sensory function. This could offer unique therapeutic potential, especially for patients with chronic pain, such as spinal cord injury patients, who usually have diminished sensory function.

This pattern is notable given the multidimensional nature of pain, which comprises interacting sensory-discriminative, affective-motivational, and cognitive-evaluative components [2]. The preservation of sensory encoding coupled with reduced cognitive–affective evaluation suggests that BreEStim modulates the transformation of nociceptive input into subjective pain experience, consistent with predictive coding and salience-based models of pain perception [46].

The induction of a distinct resting-state cortical configuration further suggests that BreEStim effects may extend beyond immediate analgesia, potentially supporting sustained modulation of baseline pain processing. Repeated sessions may therefore promote activity-dependent neuroplastic changes, an effect enhanced by the active engagement of breathing and attentional control, which aligns with the contemporary emphasis on patient agency and self-management in chronic pain care [6,47].

Clinically, BreEStim may offer several advantages as a non-pharmacological intervention that is amenable to self-administration after training and combines peripheral stimulation with a low-cost behavioral component. Its efficacy likely reflects integrated modulation of cortical pain networks, respiratory–autonomic coupling, and descending inhibitory mechanisms, potentially providing multiple pathways for therapeutic benefit.

### 4.5. Limitations and Future Directions

Several limitations should be acknowledged. First, the sample consisted of healthy participants exposed to experimentally induced pain rather than chronic pain patients. While this controlled approach allows clear characterization of BreEStim’s neurophysiological effects, translation to clinical populations requires confirmation that similar mechanisms operate in chronic pain states where neuroplastic changes may have altered baseline cortical organization. Second, the single-session design captures acute effects but cannot address whether repeated BreEStim produces cumulative neuroplastic changes or sustained analgesic benefits as seen with meditation [47]. Longitudinal studies with chronic pain patients are needed to establish the durability of cortical modulation and its relationship to clinical outcomes.

Third, the behavioral outcomes were assessed within 3 min after the intervention, whereas in our previous studies, measurements were obtained after a longer post-intervention interval. This may influence the interpretation of the observed threshold changes as an analgesic effect and limits direct comparison with earlier findings. In addition, EPT was not measured during the intervention, preventing characterization of the temporal evolution of pain modulation and the precise onset of threshold changes.

Although we interpret P250 as reflecting anterior cingulate cortex engagement, EEG has limited spatial resolution compared to neuroimaging methods. Future studies combining EEG with functional MRI or magnetoencephalography could provide more precise localization of neural generators. Moreover, the specific breathing parameters were based on prior behavioral protocols and were not systematically varied. Parametric manipulation of breathing rate, depth, and stimulation timing could optimize the intervention and clarify which aspects of respiratory coupling are most critical for pain modulation.

Furthermore, while breathing-only control has been studied previously [23], future studies may also include it to explore the neurological mechanisms related to breathing only. Further research should also examine whether the identified cortical mechanisms generalize across different pain modalities and chronic pain conditions, as pain etiology may influence responsiveness to modulation. Additionally, exploring potential synergistic effects between BreEStim and complementary interventions, such as cognitive-behavioral therapy or mindfulness-based approaches, may help optimize its therapeutic efficacy.

## 5. Conclusions

This study provides the first detailed characterization of the cortical mechanisms underlying BreEStim-induced pain modulation. By demonstrating selective reduction in affective pain processing (Cz P250 attenuation) while preserving early sensory responses (P30), and by revealing distinctive alterations in resting-state cortical dynamics (reduced alpha band power), our findings indicate that BreEStim operates primarily through higher-order modulation of pain evaluation and salience attribution rather than peripheral sensory gating. As compared with the other modalities discussed above, these BreEStim-specific neuromodulatory mechanisms support BreEStim’s development as an evidence-based, non-pharmacological intervention for chronic pain conditions, particularly those characterized by heightened affective suffering and cognitive elaboration of pain.

## Figures and Tables

**Table 1:** Summary of the three-way repeated-measures ANOVA examining the effects of Intervention (BreEStim vs. EStim), Time (pre vs. post), and Hand (right vs. left) on pain threshold, including F-values, *p*-values, and partial eta-squared effect size. Significance levels are indicated as ** *p* < 0.01, *** *p* < 0.001.

Effects	F	*p*	ηp^2^
Intervention	0.216	0.650	0.016
Time	11.382	0.005 **	0.467
Hand	1.525	0.239	0.105
Intervention × Time	0.023	0.881	0.002
Intervention × Hand	0.462	0.509	0.034
Time × Hand	55.542	0.001 ***	0.810
Intervention × Time × Hand	0.121	0.734	0.009

**Table 2:** Summary of two-way repeated measures ANOVAs for different ERP waveform components examining the effects of Intervention (BreEStim vs. EStim) and Time (pre vs. post) on peak amplitudes, including F-values, *p*-values, and partial eta-squared effect sizes. Significance levels are indicated as * *p* < 0.05.

Effect	P30, CP3			P250, CP3			P250, Cz		
	F	*p*	ηp^2^	F	*p*	ηp^2^	F	*p*	ηp^2^
Intervention	0.007	0.933	0.001	1.924	0.189	0.129	0.141	0.714	0.012
Time	1.790	0.204	0.121	5.734	0.032 *	0.306	6.477	0.026 *	0.351
Intervention × Time	0.095	0.762	0.007	0.123	0.731	0.009	7.237	0.020 *	0.376

**Table 3:** Summary of two-way repeated measures ANOVA for different frequency bands for DLPFC activity, examining the effects of Intervention (BreEStim vs. EStim) and Time (pre vs. post) on average activity over F3 and F4, including F-values, *p*-values, and partial eta-squared effect sizes. Significance levels are indicated as * *p* < 0.05, ** *p* < 0.01.

Effect	Delta			Theta			Alpha			Beta		
	F	*p*	ηp^2^	F	*p*	ηp^2^	F	*p*	ηp^2^	F	*p*	ηp^2^
Intervention	5.702	0.033 *	0.305	0.456	0.511	0.034	6.644	0.023 *	0.338	1.075	0.319	0.076
Time	0.607	0.450	0.045	5.806	0.032 *	0.309	1.622	0.225	0.111	0.248	0.627	0.019
Intervention × Time	2.717	0.123	0.173	0.670	0.428	0.049	11.71	0.005 **	0.474	1.683	0.217	0.115

## Data Availability

Data can be made available upon request from the corresponding author.
